# A randomized, double-blind, placebo-controlled trial on the role of preemptive analgesia with acetaminophen [paracetamol] in reducing headache following electroconvulsive therapy [ECT]

**DOI:** 10.1186/s12888-017-1444-6

**Published:** 2017-07-28

**Authors:** Amila Isuru, Asiri Rodrigo, Chamara Wijesinghe, Dileepa Ediriweera, Shan Premadasa, Carmel Wijesekara, Lalith Kuruppuarachchi

**Affiliations:** 1 Kingswood estate, Paravahera, Kekanadura, Matara, Sri Lanka; 2 University Psychiatry Unit, Colombo North Teaching Hospital, Ragama, Sri Lanka; 3 Department of Psychiatry, Faculty of Medicine, University of Kelaniya, Ragama, Sri Lanka; 4 Centre for Health Informatics, Biostatistics and Epidemiology, Faculty of Medicine, University of Kelaniya, Ragama, Sri Lanka; 5 Colombo North Teaching Hospital, Ragama, Sri Lanka

**Keywords:** Headache, Electro convulsive therapy, Preventive analgesia, Preemptive analgesia, Acetaminophen, Predictors

## Abstract

**Background:**

Electroconvulsive therapy (ECT) is a safe and efficient treatment for several severe psychiatric disorders, but its use is limited by side effects. Post-ECT headache is one of the commonest side effects. Preemptive analgesia is effective in post-surgical pain management. The most commonly used analgesic is acetaminophen (paracetamol). However, acetaminophen as a preemptive analgesic for post-ECT headache has not been studied adequately. This study was conducted to compare the incidence and severity of post-ECT headache in patients who were administered acetaminophen pre-ECT with a placebo group.

**Methods:**

This study was a randomised, double-blind, placebo-controlled trial. Sixty-three patients received 1 g acetaminophen and 63 patients received a placebo identical to acetaminophen. The incidence and severity of headache 2 h before and after ECT were compared between placebo and acetaminophen groups. The severity was measured using a visual analog scale. Generalised linear models were used to evaluate variables associated with post ECT headache.

**Results:**

Demographic and clinical variables of placebo and acetaminophen groups were comparable except for the energy level used to induce a seizure. Higher proportion of the placebo group (71.4%) experienced post-ECT headache when compared to the acetaminophen group (*p* < 0.001). The median pain score for headache was 0 (Inter quartile range: 0–2) in acetaminophen group whereas the score was 2 (IQR: 0–4) in placebo group (*P* < 0.001). Model fitting showed that the administration of acetaminophen is associated with less post-ECT headache (odds ratio = 0.23, 95% CI: 0.11–0.48, *P* < 0.001).

**Conclusion:**

A significant reduction was seen in both the incidence and severity of post-ECT headache with preemptive analgesia with acetaminophen.

**Trial registration:**

Ethical approval was granted by an Ethic review committee, University of Kelaniya, Sri Lanka (P/166/10/2015) and the trial was registered in the Sri Lanka Clinical Trials Registry (SLCTR/2015/27).

## Background

Electroconvulsive therapy (ECT) is one of the most effective and life-saving treatment modalities in psychiatry given its rapid response compared to pharmacological treatment [1, 2]. ECT is reserved for patients suffering from severe, treatment resistant depression, mania, schizoaffective disorder and schizophrenia. Treatment response to ECT in patients with severe depression and mania is reported to be 83% [3] and 78% [4] respectively. It is considered to be the primary treatment option for patients with suicide risk, severe self- neglect in the context of affective disorders and for catatonia and neuroleptic malignant syndrome which demands prompt treatment response [5]. There are three ways of administering ECT, out of which bilateral ECT is the most effective and commonly used method [5, 6].

In spite of its improved safety, ECT remains an underutilized treatment across the globe [7]. Stigma, side effects, poor understanding about the efficacy and misconceptions around ECT have been identified as major barriers to the appropriate use of ECT [8].

Headache following administration of ECT, one of the most common side effects, is experienced by 48% to 85% patients who undergo this treatment [9, 10]. Post-ECT headache is experienced immediately or shortly after patients regain consciousness. Sometimes it can be severe, prolonged, and remain unresolved for 2–3 days [11]. Patients who experienced this type of headache may be reluctant to accept subsequent ECT sessions [10].

Etiology of post-ECT headache is yet to be fully understood; it is postulated that ECT triggers headache due to rapid contraction of temporalis and messeter muscles, vascular changes and alteration of serotonergic neuro transmission in the brain occurring with ECT [9, 10]. Efficient management of post-ECT headache is important since it reduces patient suffering and would help to enhance favorable attitude towards ECT.

Preemptive analgesia has been identified as an effective method in preventing post-ECT headache [12]. Preemptive analgesia is based on a physiological phenomenon called central sensitization where transmission of pain signals evoked by tissue damage leads to sensitization of pain pathways [13]. Preemptive analgesia prevents central sensitization by giving the analgesia before tissue injury, thereby inhibiting the response to nociceptive stimuli. Some studies found it was more effective than a similar analgesic treatment initiated after surgery [13].

In spite of its promising potential, use of pre-emptive analgesia in the prevention of post-ECT headache has been understudied. There is only one randomized controlled study assessing the use of pre-emptive analgesia which recommended the use of Ibuprofen as a premedication in patients with post-ECT headache [12]. However, use of Ibuprofen is questionable in a patient who is fasting given its high propensity to cause gastric irritation and the drug interactions with other commonly used psychotropic medications such as lithium. Acetaminophen (paracetamol) is as effective as Ibuprofen as an analgesic and it has been recommended for preemptive analgesia in ECT [14]. However, its effectiveness in this regard has not been studied.

Therefore, we undertook a double-blind, placebo-controlled, randomized trial to assess the effectiveness of preemptive analgesia with acetaminophen in reducing the incidence and severity of a post-ECT headache.

## Methods

### Study setting

The study was carried out in the psychiatry unit of North Colombo Teaching Hospital, Sri Lanka, one of the largest tertiary care hospitals in the country with a bed strength more than 1000. ECT sessions were conducted in a dedicated ECT suite under the supervision of a consultant psychiatrist and consultant anaesthetist. Thymatron system IV (Somatics, LLC, Lake Bluff, IL, USA, Class 1, type BF) ECT machine was used in the treatment of all patients. All the study participants received bilateral ECT and the energy level was calculated according to the stimulus dosing method [15]. Propofol was used as the anaesthetic agent while suxamethonium was used as the muscle relaxant. Energy levels used in administering ECTs were decided according to the individual seizure thresholds which varied from person to person with age, gender and current medication [16]. Index episode as referred to in this research article, is the episode of current admission to the hospital for ECT.

### Subjects

All patients who were admitted for ECT and gave informed written consent were included in the study. Exclusion criteria included patients who gave a history of allergy to acetaminophen, lacked capacity to give informed consent and developed complications such as delirium following ECT. Unilateral and bi-frontal ECT recipients were excluded since those methods are practiced very rarely in our unit.

We assessed all the patients for history of headache and treatment with analgesic medications during this episode. Eleven patients gave a history of headache during this episode. No patients had received any type of analgesia in the 24 h prior to the ECT except as part of the study protocol.

### Sample size

When the sample size was calculated to detect an odds ratio of 3, assuming 45% incidence of headache in the unexposed group with 95% confidence level and 80% power and 1:1 ratio in the intervention and control groups, sample size for each group was 63 participants with a total of 126 for the study. Open Epi Version 2.3.1 was used to calculate sample size [17].

**Fig. 1:**
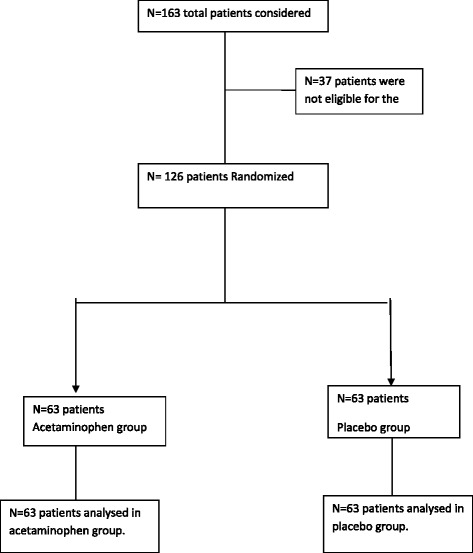
Trial profile- participants flow chart

One hundred and sixty-three patients were assessed to recruit 126 consenting participants to the study from January 2016 to July 2016 (Fig. 1). Patients demographic and clinical variables were gathered by a medical officer before the ECT.

### Randomization

Identical placebo tablets were designed by the State Pharmaceutical Manufacturing Corporation of Sri Lanka. There were 63 sealed and opaque envelopes containing two acetaminophen tablets each and 63 similar envelopes with placebo tablets. These envelopes numbered randomly from 1 to 126. Envelope number and corresponding type of tablets were recorded and kept in the investigator’s office. Sealed envelopes were given to the nurses in charge of male and female wards, who along with other ward staff were blind to the content of the envelopes. One hundred and twenty six cards numbered from 1 to 126 were kept in a box at the nurses’ stations for the purpose of randomization. On the day before each ECT, patients were randomized using these numbered cards, where the nursing officer drew a numbered card randomly from the randomization box and numbered card was attached to the bed head tickets of the patients. On the following day, relevant sealed envelopes were obtained from the nurse in charge and concealed tablets given to the patients 2 h prior to the ECT. Once the tablet was given, numbered card was detached from the bed head ticket and discarded to ensure randomization without replacement. Therefore, all the patients received either two 500 mg tablets of acetaminophen or two placebo tablets which were identical to the acetaminophen tablets, 2 h before the general anaesthesia.

### Assessment of headache

All the patients enrolled in the study were assessed for a headache 2 h before and after the ECT. Headache assessments were done by independent assessors who were medical officers working in the psychiatry unit and they were kept blind on the placebo or acetaminophen status of the patients. Following the inquiry, severity of headache was evaluated using a visual analog test with zero (0) labelled as no pain and ten (10) labelled as an excruciating headache.

### Statistical analysis

Group comparisons with respect to interventional and control were done using Pearson’s Chi-square test and Fisher’s exact test for categorical data, and Mann Whitney U test for ordinal data and continuous data when normality was not present. New occurance of headache in those who previously did not have headache or worsening of headache in those who already had headache before giving ECT, were considered as positive and others as negative in modelling post ECT headache.

Initially, patient’s demographic and clinical variables that may affect the post ECT headache (exposure variables) were screened with Pearson’s Chi-square test for post ECT headache. Subsequently, exposure variables significant at *p* = 0.2 were selected for linear logistic model fitting considering post ECT headache as the response variable. Adequacy of the fitted model was assessed with Hosmer and Lemeshow goodness of fit test [18]. Since the fitted model showed overdispersion (i.e. fitted model was not adequate to represent the response variable), a generalized linear model was fitted considering quasibinomial distribution and parameters were estimated [19]. The difference between the levels of the exposure variable was reported with odds ratio, confident interval of the odds ratio and *p* value. A *p* value of 0.05 was considered as significant. Analysis was performed using R programming language version 3.2.3 [20].

## Results

### Sample characteristics

The majority (58.7%) was females and 43.7% of the sample was in the age range of 41–60 years, followed by the range of 18–40 years (39.7%). The commonest diagnosis in the study population was depression (42.9%), followed by bipolar affective disorder (31%). Eleven patients (8.7%) had experienced headache prior to episode and 7.1% of participants gave a history of migraine.

Demographic, clinical and ECT-related parameters were comparable between placebo, and treatment groups at the baseline except for the energy level used to induce seizure (Table 1). There was no statistically significant difference in median severity of headache before the ECT in acetaminophen and placebo groups (median severity of headache was 0 in both groups). Use of antidepressants, antipsychotics, mood stabilizers, benzodiazepines and medications used in anaesthesia (propofol and suxamethonium) were not significantly different in both groups.

**Table 1:** Characteristics of treatment and placebo group

Variable		(Acetaminophen) Paracetamol	Placebo	*P* value
Age	18-40 yrs	24	26	0.71^a^
	41-60 yrs	28	27	0.85^a^
	>60 yrs	11	10	0.81^a^
Sex	Male	21	31	0.07^a^
	Female	42	32	
Marital status	Married	28	29	0.60^a^
	Single	35	34	
Number of patients use alcohol	Alcohol dependent patients	5	4	0.15^b^
	Alcohol misuse patients	9	18	
Number of patients do not use alcohol		49	41	
Diagnosis	Depression	32	22	0.07^a^
	Bipolar affective disorder	17	22	0.33^a^
	Schizophrenia with secondary depression.	14	19	0.46^a^
Clinical Parameters	Comorbid medical illness	19	14	0.31^a^
	Migraine	4	5	1.00^b^
	Headache during this episode	4	7	0.52^b^
ECT-related parameters.	Seizure duration Median (IQR)	20 (15–30)	20 (15–30)	0.94^c^
	Energy level Median (IQR)	10 (5–10)	10 (10–10)	<0.01^c^

^a^chi-square test, ^b^Fishers exact test, ^c^Mann Whitney U test; *IQR* Interquartile range

It is very unlikely that patients received analgesic medications 10 h prior to ECT as they were kept fasting during that time. Acetaminophen received prior to that would have little impact on post-ECT headache due to its short half-life.

**Fig. 2:**
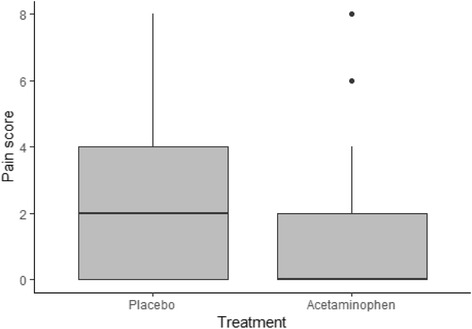
Boxplot of headache pain scores 2 h after ECT between placebo and acetaminophen group

The median post-ECT headache pain score of acetaminophen group (median = 0; inter quartile range [IQR] = 0–2) was significantly lower than the placebo group (median = 2; IQR = 0–4) (*p* < 0.001) (Fig. 2).

The incidence of post ECT headache was 36% (23/63) in patients who received acetaminophen compared to 71% (45/63) in the placebo group (Table 2). Significantly lower incidence of post ECT headache in the acetaminophen group compared to placebo group (*p* < 0.001) may suggest effectiveness of acetaminophen in pre-emptive treatment of post-ECT headache. Hence, the number needed to treat (NNT) or the average number of patients needed to treat to prevent one additional post-ECT headache is 3.0.

**Table 2:** Headache score and number perceived headache before giving ECT vs after receiving ECT

	Pre ECT	Post ECT
	Median headache score (IQR)	Median headache score (IQR)
Placebo group	0 (0–0)	2 (0–4)
PCM group	0 (0–0)	0 (0–2)
	Participants perceived headache (%)	Participants perceived headache (%)
Placebo group	7 (11%)	45 (71%)
PCM group	4 (6%)	23 (36%)

Inter quartile range (IQR), percentage (%)

Initial variable screening showed, age group, employment, history of migranous headache, headache during episode and treatment status (i.e. acetaminophen or placebo) were significant at *P* = 0.2 level. Subsequently, multiple linear logistic model fitting showed, only the treatment status was significantly associated with post-ECT headache, and administration of acetaminophen significantly lowered post-ECT headache (odds ratio = 0.23, 95% CI: 0.11–0.48, *p* < 0.001) (Table 3). Analysis did not show any association between post-ECT headache and other possible covariates such age, sex, marital status, psychiatric diagnosis, comorbid medical illnesses, history of a migraine, headache during the index episode, energy level used to induce the seizure or the seizure duration (Table 3).

**Table 3:** Parameter estimate of the fitted model for post ECT headache

Variable	Estimate	Standard error	*t* value	*P* value
Initial model				
Intercept	5.12	2.53	2.03	<0.001
Age group: 1 vs 2	−0.09	0.44	−0.21	0.834
Age group: 1 vs 3	1.03	0.62	1.66	0.097
Employement	0.69	0.42	1.66	0.974
History of migranous headache	−0.89	0.94	−0.95	0.341
Headache during episode	−1.57	0.87	−1.80	0.0719
Acetaminophen	−1.42	0.42	−3.42	<0.001
Final model				
Intercept	0.92	0.28	3.26	<0.001
Acetaminophen	−1.47	0.39	−3.81	<0.001

Age group 1: 18 to 40 years; Age group 2: 41 to 60; Age group 3: over 60 years

## Discussion

Our study showed that acetaminophen administered two hours prior to ECT reduced the incidence and severity of post-ECT headache, one of the commonest adverse effects of ECT [21, 22]. Though we did not assess the impact of headache, it has been recognized as a cause for premature termination of the ECT [11, 23]. There is a dearth of studies investigating the effectiveness of pre-emptive analgesia for post-ECT headache. Most of them are case studies [24]. The only randomized controlled trial was using ibuprofen as a pre-emptive analgesic [12] which too demonstrated a lower incidence of post-ECT headache. In this study 17 patients who received Ibuprofen showed significantly lesser incidence and severity of headache compared to similar number of patients who received placebo treatment [12]. Furthermore, Ibuprofen has a less favorable side effect profile and more drug interactions when compared to acetaminophen. It is also noteworthy that acetaminophen does not alter seizure threshold.

The study revealed administration of 1 g acetaminophen 2 h prior to ECT was effective in reducing both incidence and severity of post-ECT headache. Improvement of headache was comparable to what was reported by Lueng et al. with preemptive treatment of ibuprofen [12].

A study done by Haghighi et al. reported 21.6% of post ECT headache [25] which is relatively lower than what we found in our study. This may be explained by the fact that in our study, post ECT headache was assessed 2 h after the ECT while the study done by Haghighi et al., assessed the headache 6 h after the ECT. In six hours the headache may have improved resulting in underreporting of headache.

In our study population only 8% of patients reported headache as part of their psychiatric episode. A study in the outpatients department of another tertiary hospital of Sri Lanka showed that 44% of patients with depressive disorder experienced headache [26]. It should be noted that our study population is heterogeneous and included patients with diagnoses other than depressive disorder.

We did not identify any predictors of post-ECT headache in relation to age, gender, marital status, history of substance misuse, medical comorbidity, energy level and the seizure duration. The study by Haghighi et al., too did not identify predictors of post ECT headache with 621 patients [25].

### Limitations

The inherent difficulty in reliably measuring pain perception using self-report scales, has to be acknowledged. There are numerous factors such as gender, age, cognitions and prevailing mood, that affect the perception of pain. Pain is subject to personal bias in reporting and interpretation [27]. Some patients may also find it difficult to indicate the subjective experience of pain in a straight line visual analogue scale [28]. This can be overcome by giving simple and comprehensive instructions regarding the visual analogue scale at the time of the interview which we did. Administration of self-report faces of Pain Scale with the visual analogue test to quantify the severity of pain more precisely could be used to improve the reliability of pain measurement. The visual analogue scale though has the advantage of reliably measuring pain across different cultures [29, 30].

We also did not assess headache beyond 2 h after ECT. It is unlikely though that the headache could have emerged thereafter. The presence of headache prior to ECT or having a history of migraine did not appear to influence post ECT headache. The smaller number of patients with the above mentioned conditions could have contributed to the negative finding The acetaminophen group received significantly higher energy level than the placebo group. If at all, this should have contributed to a higher likelihood of headaches, although previous studies have also shown that the energy level used to induce seizure do not predict post-ECT headache [25].

## Conclusions

This is the first study to assess the effectiveness of acetaminophen (paracetamol), a cheap and freely available analgesic, as a preemptive analgesic for post-ECT headache. It is worthwhile considering the inclusion of these findings in ECT guidelines for routine practise.
